# The bare head of the Northern bald ibis (*Geronticus eremita*) fulfills a thermoregulatory function

**DOI:** 10.1186/s12983-017-0201-5

**Published:** 2017-03-01

**Authors:** Ismael Galván, Daniel Palacios, Juan José Negro

**Affiliations:** 1 Department of Evolutionary Ecology, Doñana Biological Station - CSIC, 41092 Sevilla, Spain; 2 Department of Zoology, University of Córdoba, 14071 Córdoba, Spain

**Keywords:** Bare skin, Heat stress, Northern bald ibis, Pigmentation, Thermal imaging, Thermal radiators

## Abstract

**Background:**

Dark pigments provide animals with several adaptive benefits such as protection against ultraviolet (UV) radiation and mechanical abrasion, but may also impose several constraints like a high absorbance of solar radiation. Endotherms, with relatively constant and high body temperatures, may be especially prone to thermoregulatory limitations if dark coloured and inhabiting hot environments. It is therefore expected that adaptations have specifically evolved because of these limitations. Bare, highly vascularised head skin may have evolved in birds with dark plumage from hot geographical regions because of favouring heat dissipation. Using the Northern bald ibis (*Geronticus eremita*) as a model species, we measured the surface temperature (T_surf_) of the head, the bill and the black feathered body of 11 birds along ambient temperatures (T_a_) ranging from 21 to 42.5 °C employing thermal imaging.

**Results:**

While T_surf_ of the bill and the feathered body was only slightly above T_a_, head T_surf_ was considerably higher, by up to 12 °C. Estimated values of heat loss followed similar variations. We also found that the red colour intensity of the head of ibises increased with head T_surf_, suggesting that birds are capable of controlling blood flow and the thermoregulatory function of the head.

**Conclusions:**

These findings are consistent with the hypothesis that bare skin has evolved in dark pigmented birds inhabiting hot environments because of their ability to dissipate heat.

**Electronic supplementary material:**

The online version of this article (doi:10.1186/s12983-017-0201-5) contains supplementary material, which is available to authorized users.

## Background

Endotherm animals (birds and mammals) possess the capacity to use heat produced by metabolism to keep a relatively constant and favourable internal temperature and avoid complete environmental dependance. This has in turn favoured the evolution of a diversity of adaptations that help maintaining temperature homeostasis when the temperature of the environment greatly differs from metabolically acceptable limits [1]. These adaptations represent a phenotypic integration among traits [2], and do not only evolve in response to the environmental conditions to which the species are exposed, but also in response to the existence of certain intrinsic characteristics of the species that have evolved because of reasons different from thermoregulation and that constitute constraints to temperature homeostasis.

One of the species traits affecting temperature homeostasis is pigmentation, particularly that associated to black and dark brown colours typically conferred by melanins [3]. This is because darker animal colours have a greater capacity to absorbe solar radiation and then achieve more heat gain [4]. The Kirchhoff’s law of thermal radiation states that emmisivity equals absorptivity at thermodynamic equilibrium, meaning that darker animals also emit more radiation, which mostly occurs in the infrared (IR) spectral region. The ecological advantage of this phenomenon is well known in reptiles, where it is termed thermal melanism and allows darker coloured species or morphs to occupy cooler habitats [5]. By contrast, the capacity of endotherms to maintain relatively high and constant body temperatures makes them less dependent on traits that favour heat gain to occupy cool habitats than terrestrial ectotherms, as evidenced by the almost complete absence of the latter in the coldest regions on Earth [6]. Thus, a trait that confers an extra gain of heat such as a dark colouration may even represent a thermoregulatory constraint for an endotherm living in a hot environment. Indeed, some researchers consider that endothermy may be a constraint and not only an adaptation, although little knowledge on the thermosensitivity of performance in endotherms exists ([2, 7]; see however [8, 9]). As a consequence, the influence of pigmentation on the thermoregulatory behaviour of endotherms and its ecological and evolutionary implications are poorly understood and represent an underexplored field.

Only a few studies so far have investigated possible constraints that dark coloured endotherm animals must face to achieve temperature homeostasis and how they affect their ecological interactions. It has been reported that dark-maned male African lions (*Panthera leo*) acquire higher surface temperatures (T_surf_) than lighter males [10]. Hochscheid et al. [11] found that temperature in juvenile Cape gannets (*Morus capensis*), which display black plumage, is significantly higher on dorsal plumage surface and inner body than in adults, which display white plumage, when exposed to high ambient temperatures (T_a_). This forces juvenile Cape gannets to spend more time thermoregulating by evaporative cooling than adults, and as this behaviour produces water loss, total water volume available becomes limited to juveniles, which may have negative consequences for their survival in the hot environments that they inhabit [11]. Similar results have been found for the springbok (*Antidorcas marsupialis*), a medium-size antelope that inhabits hot areas of Southern Africa and that presents three pelage colour morphs of different darkness [12]. The maximum body temperature displayed by black springboks is higher than that of light brown and white springboks, which reduced the degree of diurnal activity of black animals during the hottest period (summer) as compared to the lighter morphs [12]. There is also some evidence from domestic animals that dark pigmentation reduces tolerance to heat stress, as in goats with black coat [13]. Some studies have suggested that variation in wind speed and certain behaviours such as ptiloerection makes the association between pigmentation and heat gain more complicated than described above [14, 15], but given that these studies were conducted on museum skins instead of live animals it is not possible to determine to which extent such conclusions are realistic.

Dark pelage colouration may also be an advantage because it can reduce the metabolic costs of homeothermy during cold periods, as suggested by the fact that black springboks spend less time feeding than the lighter morphs during the winter [12]. The black skin of polar bears (*Ursus maritimus*) beneath their white pelage is another example of dark pigmentation being adaptive for endotherms in cold environments or periods [16], as well as the heat gain achieved by black feather patches in bearded vultures (*Gypaetus barbatus*) that inhabit cold habitats [17] and by dark pelage bands in basking dunnarts (*Sminthopsis crassicaudata*) [18]. It has even been suggested that climate warming may be decreasing the proportion of the dark brown morph of Soay sheep (*Ovis aries*) [19]. However, the previous studies cited above provide evidence, albeit scarce, that dark pigmentation, having evolved because of different adaptive benefits (i.e., protection against UV radiation, mechanical damage or feather-degrading bacteria, and visual signaling to conspecifics or to other species; see [20] for a review), constitutes a constraint for homeothermy in hot environments.

Given such a constraint, it is expected that birds and mammals displaying dark colouration and inhabiting hot environments have evolved adaptive strategies (beyond behavioural responses such as staying in sheltered places or in the shade) to counteract overheating and facilitate physiological performance within thermal tolerance limits. Although a diversity of adaptations to high temperatures has been described for homeotherms [1], those that have specifically evolved because of the thermal constraint imposed by dark pigmentation are virtually unknown. It has been suggested that fox squirrels (*Sciurus niger*) of the black morph have thinner hairs than those of lighter morphs to facilitate air flow for evaporative cooling [21]. Beyond this specific suggestion, Negro et al. [22] conducted a comparative study of birds that led to a general hypothesis: bare and highly vascularised skin areas that facilitate blood surface circulation and heat dissipation have evolved in dark and large species inhabiting hot environments. Among the species considered in Negro et al.’s study [22] there are several large ratites such as the ostrich (*Struthio camellus*), which is in fact the largest of all living birds, most vulture species in both the New World (condors and allies) and the Old World (all *Gyps* species, for instance), and the two wild turkeys [(*Meleagris gallopavo*) and (*M. ocellata*)]. This confirmed the previous study by Buchholz [23], who by artificially reinsulating the bare head of wild turkeys obtained some evidence that this structure functions in heat dissipation, although he did not related it to the dark pigmentation of birds. These bare skin patches quickly change colour from pale to intense red in response to variations in the amount of surface blood circulation [22], thus potentially acting as controllable vascular thermal radiators similar to the large bill of some species of birds [24, 25]. Empirical evidence of the thermal benefits of vascularised bare skin areas in dark birds exposed to high temperatures, however, has never been obtained.

**Fig. 1:**
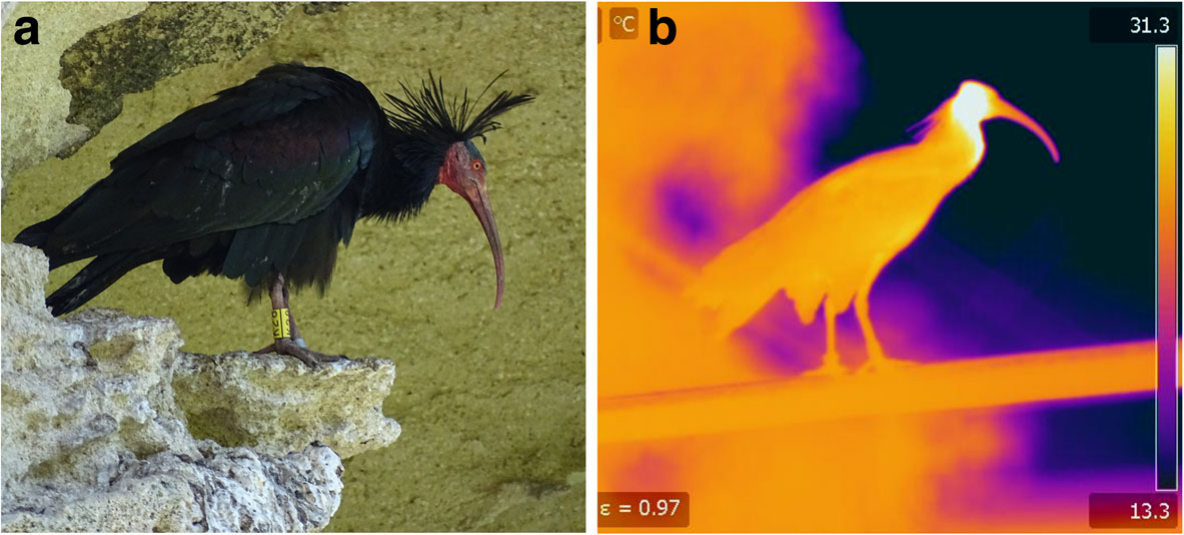
**a** a photograph of an adult Northern bald ibis (credit: JJN). **b** an IR thermal image of a Northern bald ibis, with a temperature scale on the right side. Note the contrast between the high temperature of the head and other bird regions and the environment

Our aim here is to test the above mentioned hypothesis in the Northern bald ibis (*Geronticus eremita*), a medium-sized bird that inhabits torrid areas around the Mediterranean sea [26]. Its body is entirely covered by black plumage except for the head, which presents red bare skin (Fig. 1a). Head redness intensity rapidly changes in adult birds (pers. obs.), thus suggesting that it serves as a thermal radiator to avoid overheating. The climate of the Mediterranean region is characterised by strong seasonal fluctuations in temperature, with very hot summers and temperate winters [27], which may specially favour the adaptive value of thermoregulatory devices given the greater need to dissipate heat during particular periods. We used IR thermography coupled to a long-range zoom lens to compare variations in the T_surf_ of head, bill and feathered body of Northern bald ibises living in semi-captivity across a wide range of T_a_. To test if the bare head skin of Northern bald ibises functions as a thermal radiator, we followed a procedure similar to that used to determine the thermoregulatory functionality of other bird morphological traits such as the bill of the toco toucan (*Ramphastos toco*) [24], i.e. analysing the pattern of change in temperature and heat loss across a range of T_a_.

## Methods

### Study area and animals

The study was conducted during 10 days in July 2014 at Zoobotánico Jerez (Jerez de la Frontera, Spain), where about 50 adult Northern bald ibises are kept in a large outdoor aviary (14 x 16 x 7 m) that allows birds to live in a semi-captivity regime. July is the period of the year with highest temperatures in our study area, which is within the natural distribution range of the Northern bald ibis [26]. The aviary contained a large wall of artificial rock at the bottom, and the remaining was an large plain area with dispersed vegetation, perching sites and water ponds. Measurements of the T_surf_ of birds were taken at a distance of 18 m from the wall, when birds were approximately at the same distance from the thermal imaging camera (see below). Numbered PVC rings allowed the individual identification of birds with the use of binoculars (Fig. 1a).

We obtained thermal images of 11 different birds, trying to cover a range of T_a_ as wide as possible for each bird. The final range of T_a_ considering all measurements was 21–42.5 °C (40 temperature values in 0.5 °C intervals, excepting skips between 29 and 30 °C and 40.5–42 °C), along which a total of 173 thermal images were obtained (data summarised in Additional file 1: Table S1). The high summer temperatures in the study area made impossible to obtain thermal images at T_a_ lower than 21 °C even though the measuring period was extended during the entire daylight cycle every day of the study. T_a_ was recorded to the nearest 0.5 °C with a digital hand-held thermometer beside the thermal imaging camera every time a thermal image was obtained. In addition to the head, T_surf_ measurements were taken on the feathered body area of birds for comparative purposes, as this area keeps the skin isolated from direct contact with the air and should therefore exhibit a thermal interaction with the environment different from that of the bare head skin. We also measured the temperature of the bill, as its red colour (Fig. 1a) suggests that it may also act as a thermal radiator as documented in other species [24, 25, 28]. Most measurements were taken when the birds were in shaded areas, but in some cases we could only take measurements of birds directly exposed to sunlight (all measurements at 37–42.5 °C ambient temperatures were always obtained from birds directly exposed to sunlight), but information on shade/sun exposure was controlled for in the analyses (see Statistical analyses below).

Additionally, we quantified the intensity of the red colour displayed by the head of the birds every time that a thermal measurement was obtained. For this aim we followed a simple approach consisting in categorising the intensity of the head colour of birds with a scale from 1 to 3, increasing with redness intensity as perceived by the observer with the use of binoculars. This categorisation of head colour was made by DP being unaware of the aims of the study.

### Thermal imaging

We used a 131-mm zoom lens (FLIR Systems, Wilsonville, Oregon) coupled to a FLIR SC660 thermal imaging camera (FLIR Systems), which provided images at a 640 × 480 pixel resolution. The camera was mounted on a tripod, always operating with protection from sun exposure. All images were taken on lateral views of the birds (Fig. 1b). The images were analysed with FLIR Tools software (FLIR Systems), manually selecting the area covered by the bill, the head and the feathered body and obtaining the mean temperature in the selected areas. The head area was delimited by tracing a straight line from the base of the throat (i.e., the inflection point at the intersection between the throat and the head; Fig. 1b) perpendicular to the vertical axis of the neck and another straight line from the base of the lower mandible to the base of the upper mandible. The legs were not considered in the study.

### Calculation of heat loss

Following previous studies on T_surf_ of birds [24, 29], heat loss was calculated as the sum of radiative heat exchange (Q_r_) and convective heat exchange (Q_c_) for each body region, using the following equations:

$$ {Q}_r=\varepsilon \sigma A\left({T}_{surf}^4-{T}_a^4\right) $$

$$ {Q}_c={h}_c A\left({T}_{surf}-{T}_a\right) $$

where ε is the combined emissivity of the bird and the environment (assumed to be 0.97), σ is the Stephan-Boltzman constant, A is the surface area of the body region and h_c_ is the convective heat transfer of the body region. For the calculation of the area of the three body regions considered, it was assumed that the bill was a cylinder and the head and the feathered body were spheres [29], using a bill length of 13.18 cm corresponding to the mean length reported by Siegfried [30] for different populations of the Northern bald ibis, and head and body diameters of 10 cm and 75 cm, respectively (information obtained from a dead specimen conserved at Zoobotánico Jerez).

h_c_ was calculated with the following equation:

$$ {h}_c=\frac{N_u \cdot k}{D} $$

where D is the height of the body region (from the ibis specimen we estimated that it was 1.31 cm for the bill), k is the thermal conductivity of air (*k* = 0.0241 + 7.5907*e* ^− 6^ *T*_*a*_) and N_u_ is the Nusselt number, given by the following equation:

$$ {N}_u= c{R}_e^n $$

where c and n are constants (0.615 and 0.466, respectively) and R_e_ is given by:

$$ {R}_e=\frac{V \cdot D}{\upsilon} $$

where V is air velocity (assumed to be 1 m · s^−1^, as although we did not measure it, wind was virtually absent during the study period), D is the height of the body region and υ is the kinematic viscosity of air (*υ* = − 1.088*e* ^− 5^ + 8.85*e* ^− 8^ *T*_*a*_).

### Statistical analyses

General linear mixed models were used to test for associations between head colour and temperature, where redness category was the response variable, head temperature a covariate and bird identity was added as a random factor (using the Satterthwaite method to calculate degrees of freedom in SAS software). A similar procedure was followed to test for differences in the mean T_surf_ and the mean temperature differentials (T_surf_ - T_a_) between body regions, adding body region as a fixed factor and using Tukey post hoc tests when differences were significant. To investigate variation in the T_surf_ of the different body regions, we plotted T_surf_ - T_a_ against T_a_, and this variation was analysed by means of repeated-measures ANOVA’s where T_a_ interval (in 1 °C increments) was a within-subjects factor. Tukey Unequal N HSD post hoc tests were conducted to identify significant differences in T_surf_ - T_a_ values between T_a_ intervals. All analyses were conducted with all data pooled and also excluding data obtained from birds directly exposed to sunlight. Means ± standard errors (se) are shown.

## Results

### Relationship between head colour and temperature

**Fig. 2:**
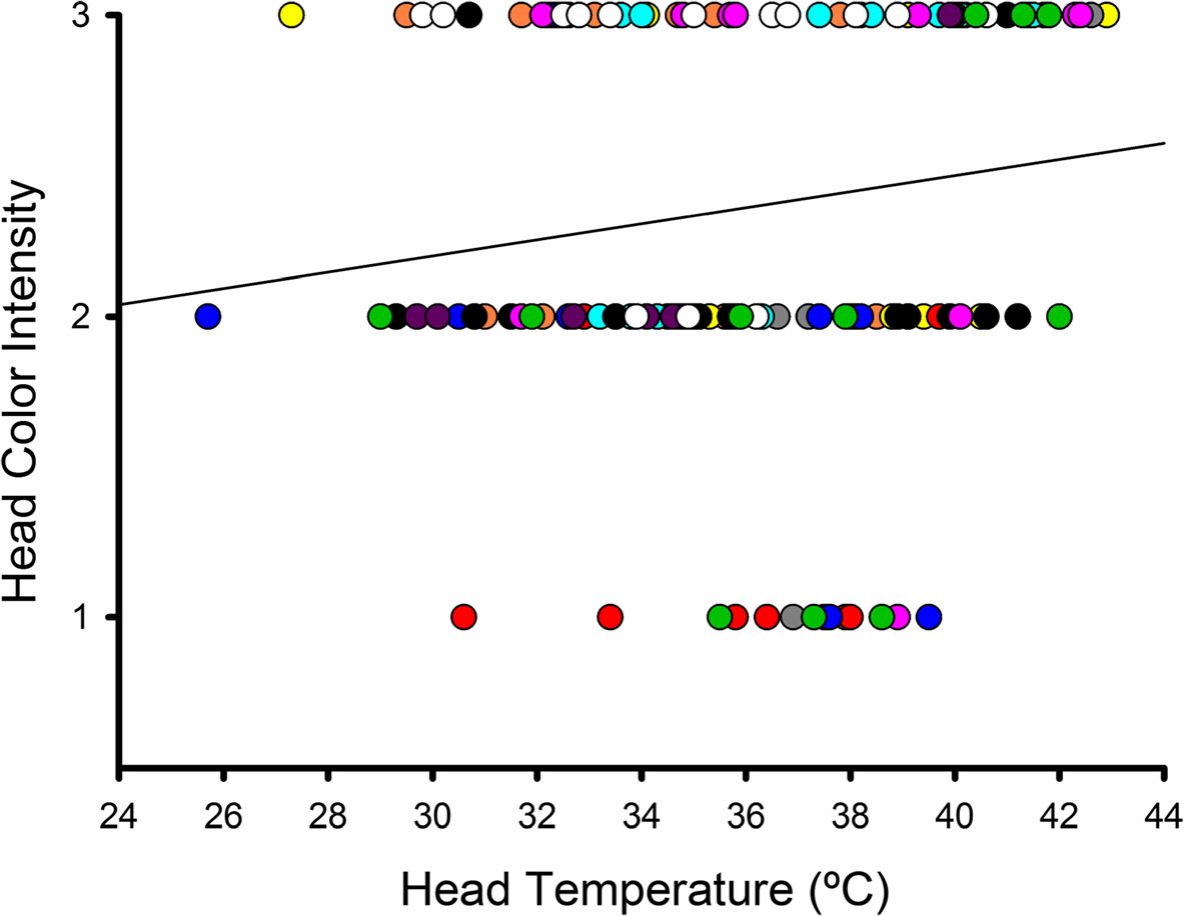
Relationship between the perceived red colour intensity of the bare head skin of Northern bald ibises and T_surf_ of the head. Red colour intensity is quantified in a scale from 1 to 3. Each symbol colour corresponds to one of the 11 birds that were included in the study. The line is the regression line

Considering all measurements taken on the 11 Northern bald ibises together, head redness category was significantly and positively related to the T_surf_ of the head (*b* = 0.029, *F*_1,141_ = 4.83, *P* = 0.029; bird identity: *P* = 0.032; Fig. 2). This association became stronger when measurements taken on birds directly exposed to sunlight were excluded from the analysis (*b* = 0.027, *F*_1,118_ = 7.16, *P* = 0.009; bird identity: *P* = 0.059). Thus, the head skin colour of Northern bald ibises reflects variation in its temperature.

### T_surf_ of bird body regions

**Fig. 3:**
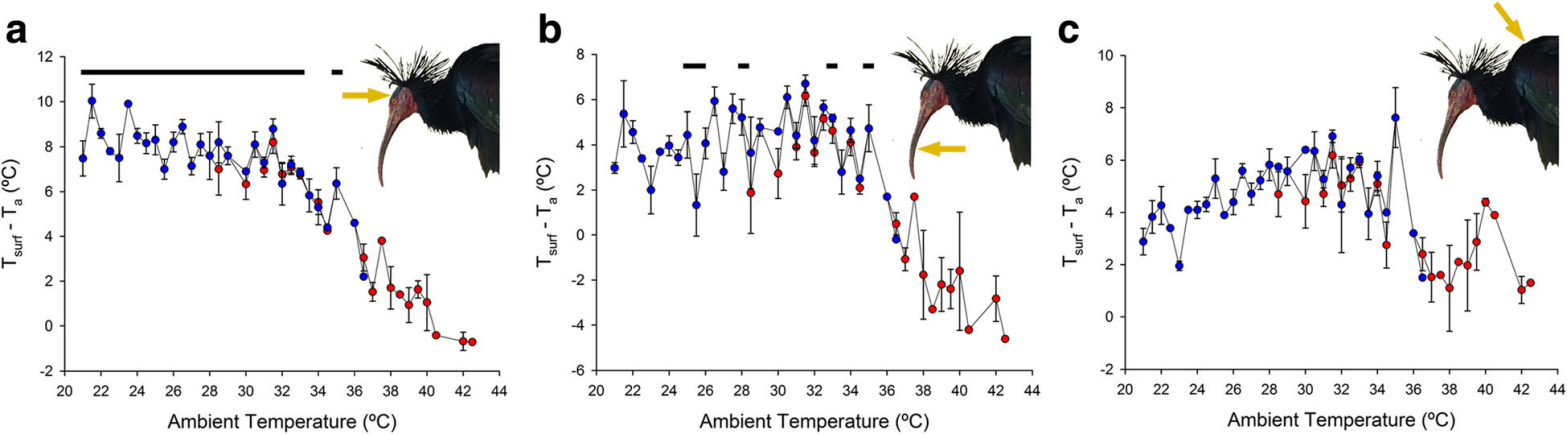
Variation in the difference between T_surf_ and T_a_ of Northern bald ibises. Data are shown in 0.5 °C intervals along T_a_ values ranging from 21.0 to 42.5 °C in the head (**a**), the bill (**b**) and the black feathered body (**c**). Symbols represent mean ± s.e. Red symbols include measurements taken on birds in the shade and directly exposed to sun light, while blue symbols only include measurements on birds in the shade. Symbols without bars indicate that only one measurement could be obtained for that specific T_a_. Horizontal black bars above graphs represent the range in T_a_ for which the mean T_surf_ - T_a_ values are significantly different from the mean T_surf_ - T_a_ in the window of 37–42 °C T_a_

The maximum T_surf_ was meaured for the black feathered body of birds, which ranged from 21.8 to 45.5 °C, while the T_surf_ of the bare head ranged from 25.7 to 42.9 °C and that of the bill from 23.0 to 42.5 °C. The maximum T_surf_ was reached at a T_a_ of 35 °C in the three body regions. However, the highest mean T_surf_ value obtained was for the head (mean ± s.e.: 36.2 ± 0.3 °C), which was significantly higher (*F*_2,506_ = 24.17, *P* < 0.0001; bird identity: *P* = 0.066) than the mean bill T_surf_ (32.8 ± 0.4 °C; post hoc test: *P* < 0.0001) and the mean feathered body T_surf_ (34.0 ± 0.4 °C; *P* < 0.0001). The mean T_surf_ - T_a_ was also significantly higher in the bare head (range: -1.4–12.0 °C; mean: 6.7 ± 0.2 °C; *F*_2,505_ = 79.00, *P* < 0.0001; bird identity: *P* = 0.043; Fig. 3a) than in the bill (range: -6.4–8.9 °C; mean: 3.3 ± 0.2 °C; *P* < 0.0001; Fig. 3b) and in the feathered body (range: -2.6–10.5 °C; mean: 4.5 ± 0.2 °C; *P* < 0.0001; Fig. 3c).

### Variation of bird T_surf_ with T_a_

T_surf_ - T_a_ of the bare head significantly varied along the range of T_a_ (*F*_20,40_ = 16.01, *P* < 0.0001; Fig. 3a), which remained marginally non-significant when measurements obtained from birds directly exposed to sunlight were excluded (*F*_14,28_ = 2.04, *P* = 0.053). T_surf_ of the head was considerably higher than T_a_ up to a limit as higher as T_a_ 35 °C, above which T_surf_ - T_a_ dropped to around zero. This was confirmed by the results of post hoc tests comparing the mean T_surf_ - T_a_ at different T_a_ intervals with the mean T_surf_ - T_a_ at the highest window of T_a_ (37–42 °C; Fig. 3a).

T_surf_ - T_a_ of the bill also varied significantly along the range of T_a_ (*F*_20,40_ = 6.36, *P* < 0.0001), but less markedly than in the head, and bird T_surf_ was only slightly above T_a_ (Fig. 3b). Indeed, this variation was no longer significant when measurements of direct sunlight exposure were excluded (*F*_14,28_ = 1.41, *P* = 0.212). Post hoc tests revealed that bill T_surf_ - T_a_ values only differed from the mean T_surf_ - T_a_ in the interval 37–42 °C of T_a_ at a few values of T_a_ (Fig. 3b).

Lastly, the results for the black feathered body showed that T_surf_ - T_a_ in this body region significantly fluctuated along the range of T_a_ (*F*_20,40_ = 4.37, *P* < 0.0001) even when measurements of direct sunlight exposure were excluded (*F*_14,28_ = 4.33, *P* < 0.001). This is because, although the black plumage of Northern bald ibises reached high T_surf_ and was considerably hotter than the ambient at medium T_a_ (around 32 °C), T_surf_ - T_a_ remained relatively constant along most part of the range of T_a_ (Fig. 3c). Indeed, T_surf_ - T_a_ of the black plumage did not differ at any T_a_ interval from the mean value in the 37–42 °C window.

### Heat loss

**Fig. 4:**
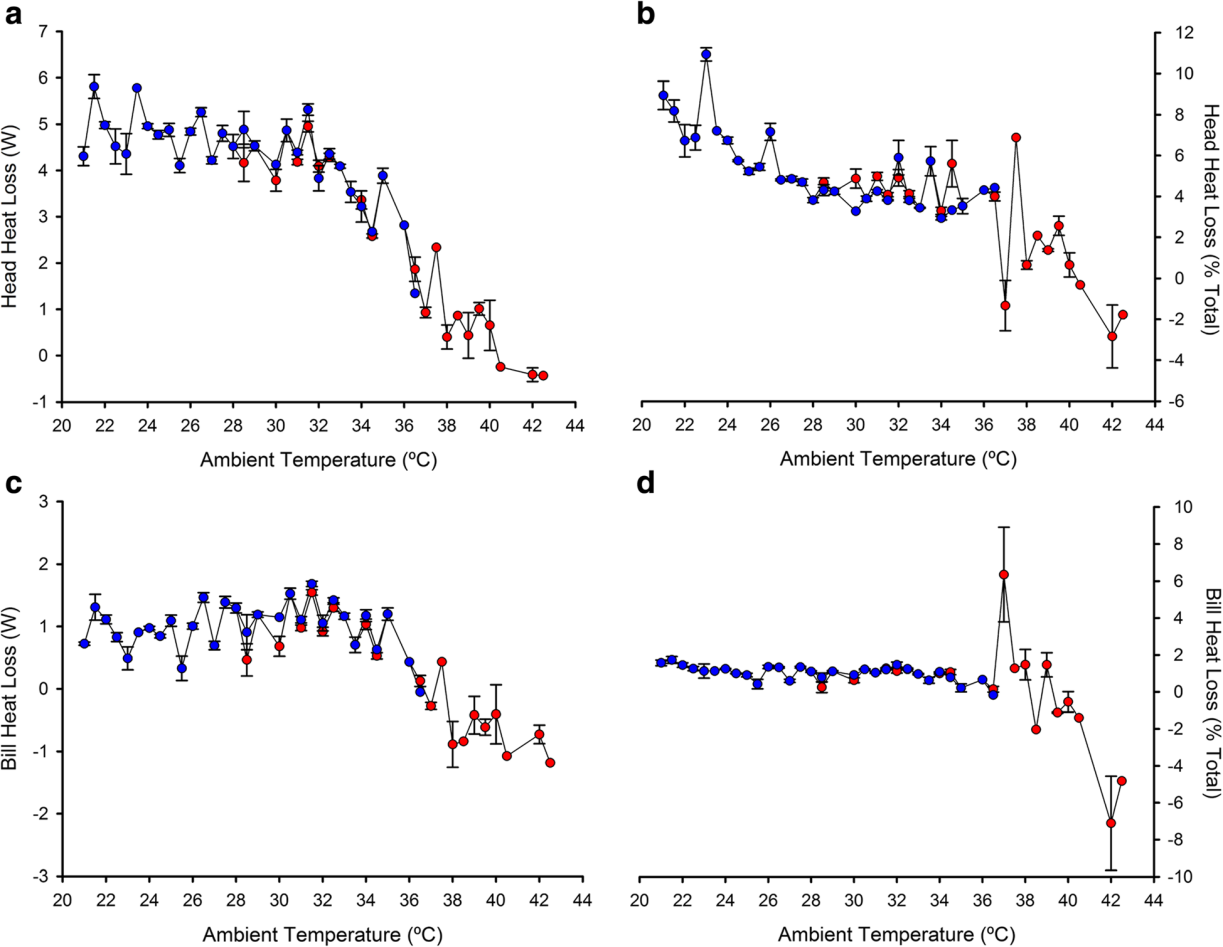
Variation in estimated heat loss for the bare head skin (**a**, **b**) and the bill (**c**, **d**) of Northern bald ibises along a range of T_a_. Both absolute heat loss values and percentage of total heat loss (the summed heat loss of head, bill and feathered body) are shown for each body region. Symbols represent mean ± s.e. Red symbols include measurements taken on birds in the shade and directly exposed to sun light, while blue symbols only include measurements on birds in the shade

As the T_surf_ of the head and the bill exhibited significant fluctuations with T_a_ (especially the head, see above), we explored the variation in the estimated heat loss for head and bill along the range of T_a_ (Fig. 4). Northern bald ibises lost considerable heat amounts through the unfeathered head, with a maximum value of 7.1 W (Fig. 4a). Accordingly with results of head T_surf_ - T_a_ values, heat loss values remained high up to a limit of T_a_ 35 °C, where heat loss dropped to zero (Fig. 4a). Similar results were obtained when heat loss was expressed as percentage of total heat loss (i.e., summed heat loss of bill, head and feathered body), as head heat loss represented 25.7% at maximum but was near zero above 35 °C of T_a_ (Fig. 4b). By contrast, heat loss through the bill fluctuated around zero along the entire range of T_a_ (Fig. 4c,d).

## Discussion

Our results show that the temperature of the bare skin of the head of Northern bald ibises is considerably higher than their environment at medium and high T_a_. The difference in temperature between the head and the environment reached 12 °C, thus representing a greater difference than that reported for the bill of the toco toucan (~10 °C), which is considered one of the largest animal thermal windows [24]. The temperature of the bill and the black feathered body, by contrast, did not differ much from T_a_ through most of the range of T_a_ considered here. The black plumage of birds, however, reached the highest T_surf_ values (45.5 °C). Thus, these findings suggest that Northern bald ibises use the bare skin of their heads to thermoregulate, therefore consistent with the hypothesis by Negro et al. [22] that black plumage pigmentation has favoured the evolution of unfeathered head structures that help dissipating heat in birds inhabiting hot environments.

The histological examinations conducted by Negro et al. [22] on the bare head skin of different species of black pigmented birds showed that these structures present a high density of peripheral blood vessels as compared to the surrounding skin covered by feathers, which indicates that this is a morphological adaptation that has specifically evolved because of its capacity to dissipate heat in species constrained by the high T_surf_ reached by their dark body regions. To our knowledge, our findings are the first empirical evidence of the functionality of an adaptive trait probably evolving because of thermal constraints imposed by dark pigmentation. On this regard it is worthy to mention that, while our measurements of T_surf_ - T_a_ for the head of the Northern bald ibis were greater than those reported for the toucan’s bill (see above), the proportion of total heat loss represented by the head of the ibises is far lower than the maximum reported for the toucans’ bill (400%; [24]). Although several factors prevent a direct comparison between these studies, we believe that the relative low (in relation to the toucan’s bill) proportion of total heat loss obtained for the head of ibises may be related to the very high temperatures reached by their black plumage surface, which made that this body region accounted for most of the heat loss. Our study was conducted outdoors with birds living in a semi-captivity regime, while Tattersall et al.’s study [24] on toucans was conducted on environmental chambers that did not go over 35 °C. Thus, it is possible that the plumage of Northern bald ibises accounted for such high proportion of total heat loss because of the easiness with which dark-coloured plumage absorbs solar radiation at the high T_a_ values in our study.

As expected, the performance of the thermal radiator had some limits, as both T_surf_ - T_a_ and heat loss of the head of birds fluctuated around zero beyond a T_a_ of 35 °C. Indeed, the maximum head T_surf_ was obtained at 35 °C of T_a_, suggesting that the functionality of the head thermal device was saturated at this value and was not useful at very high T_a_. In fact, it is expected that the amount of blood able to circulate through the head skin vessels is limited and the head is left to thermal inertia when this limit is reached [1]. The high T_a_ reached in our study area thus allowed us to determine the upper limit for the performance of the bare head skin of Northern bald ibises at 35 °C, which constitute one of the few examples reported for an animal thermal radiator. Interestingly, a recent study reporting evidence of a thermoregulatory function of the bill of hornbills considering a wide range of T_a_ (15–45 °C) also found a decrease in the temperature difference of the bill above a T_a_ around 35 °C [25]. Future studies should consider the possibility that T_a_ around 35 °C represent a generalised thermal constraint for birds.

At this stage we cannot determine whether the bare head skin of Northern bald ibises functions as a thermal radiator that is controllable by birds or if a physiological response makes increase linearly the blood flow rate in this structure with increasing T_a_ up to a limit of about 35 °C. Our limitation to answer this question is undoubtly given by our inability to obtain thermal measurements at T_a_ below 21 °C, thus preventing us from investigating the thermal behaviour of the naked head of birds at low temperatures. However, we found that the redness intensity of the bare head skin of the Northern bald ibises, measured using a simple categorisation of three values, increased with the head T_surf_. Lapped-faced vultures (*Aegypius tracheliotos*), which present dark brown plumage and bare head skin, apparently have the ability to rapidly and voluntarily change the red colour intensity of their naked heads and use it as a signal in social contests [31]. We have not investigated the potential signalling function of red skin colouration in the Northern bald ibises, but this is certainly a possibility because these birds frequently use their heads in ritualised movement displays during contests with conspecifics (pers. obs.). As T_surf_ of the head of Northern bald ibises changes with head colour intensity, it is therefore likely that these birds can control the temperature of their heads in response to variations in T_a_. This possibility should be investigated.

## Conclusions

In conclusion, our study provides the first evidence that the bare head skin that has evolved in dark pigmented birds inhabiting hot environments [22] functions as a thermal radiator in at least one species with these characteristics, the Northern bald ibis. Our findings are consistent with the hypothesis that dark pigmentation represents a thermal constraint for endotherm animals that live in hot regions because of the ability of dark integumentary structures to absorb solar radiation, and as a response highly vascularised, bare head skin has evolved as an adaptive morphological device because of its ability to dissipate heat. The ecological and evolutionary implications of possessing dark pigmented integument should also be investigated in birds and mammals in relation to the thermal constraints imposed by such pigmentation in hot environments. These studies should consider possible physiological consequences of dark animals exposed to heat stress. Although higher T_surf_ values should not necessarily be associated with higher internal body temperatures as reported in black- and white-coated Arabian camels (*Camelus dromedarius*) [32], maintaining temperature homeostasis despite higher surface temperature may constitute a physiological cost for dark animals. Studies on black domestic sheep and goats suggest the existence of such physiological consequences derived from heat stress [13, 33]. These physiological consequences would be responsible for any effects on fitness, and indeed dark-maned male lions, which suffer higher temperatures than lighter-maned lions, also show abnormal sperm and low food intake during hot periods [10]. Sirkiä et al. [34] observed that the breeding success of black male pied flycatchers (*Ficedula hypoleuca*) was the highest when T_a_ was low during the period of egg-laying, which was not observed in lighter (brown) males. While it is unknown if these results were due to the thermoregulatory constraint of being black during hot periods, it exemplifies how useful could be considering this phenomenon in studies on the ecology and evolution of pigmentation of animals including humans.

## Additional file

Additional file 1: Dataset used in the study. (XLSX 56 kb)
